# Vascular endothelial growth factor‐D modulates oxidant–antioxidant balance of human vascular endothelial cells

**DOI:** 10.1111/jcmm.13045

**Published:** 2016-12-13

**Authors:** Izabela Papiewska‐Pająk, Aneta Balcerczyk, Emilia Stec‐Martyna, Wiktor Koziołkiewicz, Joanna Boncela

**Affiliations:** ^1^ Institute of Medical BiologyPolish Academy of ScienceLodzPoland; ^2^ Department of Molecular BiophysicsUniversity of LodzLodzPoland; ^3^ Central Scientific LaboratoryMedical University of LodzLodzPoland; ^4^ Department of Cytobiology and ProteomicsMedical University of LodzLodzPoland

**Keywords:** vascular endothelial growth factor‐D, endothelial cells, redox homoeostasis, mTOR

## Abstract

Vascular endothelial growth factor‐D (VEGF‐D) is an angiogenic and lymphangiogenic glycoprotein that facilitates tumour growth and distant organ metastasis. Our previous studies showed that VEGF‐D stimulates the expression of proteins involved in cell–matrix interactions and promoting the migration of endothelial cells. In this study, we focused on the redox homoeostasis of endothelial cells, which is significantly altered in the process of tumour angiogenesis. Our analysis revealed up‐regulated expression of proteins that form the antioxidant barrier of the cell in VEGF‐D‐treated human umbilical endothelial cells and increased production of reactive oxygen and nitrogen species in addition to a transient elevation in the total thiol group content. Despite a lack of changes in the total antioxidant capacity, modification of the antioxidant barrier induced by VEGF‐D was sufficient to protect cells against the oxidative stress caused by hypochlorite and paraquat. These results suggest that exogenous stimulation of endothelial cells with VEGF‐D induces an antioxidant response of cells that maintains the redox balance. Additionally, VEGF‐D‐induced changes in serine/threonine kinase mTOR shuttling between the cytosol and nucleus and its increased phosphorylation at Ser‐2448, lead us to the conclusion that the observed shift in redox balance is regulated *via* mTOR kinase signalling.

## Introduction

Vascular endothelial cell growth factors (VEGFs) are the members of a subfamily of growth factors that are secreted signal glycoproteins produced by a number of different cell types, predominantly endothelial cells (ECs). They play an important role in the regulation of many processes due to their critical angiogenesis and lymphangiogenesis inducing functions 1. VEGFs are attractive therapeutic targets for blocking the growth of blood vessels and lymphatics in tumours, to stop the growth and spread of cancer 2.

The VEGF family currently contains seven members (A through F and PlGF) that have a common VEGF homology domain. Our interest focuses on VEGF‐D as its expression is often correlated with tumour metastasis and poor patient outcome. The expression level of VEGF‐D can serve as a marker during the metastatic processes of various cancers, including breast, bladder and colorectal cancers 3, 4, 5. Under physiological conditions in the vascular system, VEGF‐D is produced by the smooth muscle cells of small arterioles 6 and not by ECs 7 but there is an evidence that VEGF‐D participates in arteriosclerotic plaque formation 8. Only tumour‐derived ECs synthesize VEGF‐D, and these cells have been found to be resistant to apoptosis, do not undergo senescence, and to exhibit constitutive expression of markers of endothelial activation and angiogenesis 7. VEGF‐D is a plausible, alternative to VEGF‐A, as a mediator of tumour angiogenesis 9, 10. VEGF‐D signalling pathways could be important targets for new antitumour therapies; however, the mechanism underlying the biological effect of VEGF‐D on ECs is poorly understood.

Our previous study 11 focused on the autocrine effect of VEGF‐D on EC biology. VEGF‐D is initially synthesized as a disulphide‐linked pre‐propeptide containing long N‐ and C‐terminal extensions that flank a central receptor‐binding VEGF homology domain. Proteolytic processing on both N‐ and C‐terminal regions leads to formation of VEGF‐D^ΔNΔC^ which has been shown to have a significant angiogenic and vasodilatory effect, whereas the full‐length form is primarily lymphangiogenic 12. We have shown previously that the transduction of human vascular endothelial cells (HUVECs) with Ad‐VEGF‐D^ΔNΔC^ increases the expression of proteins that participate in cell–matrix interactions (integrins, matrix metalloproteinases and components of the fibrinolytic system) simultaneously with the decrease of expression of some surface membrane proteins and results in increase of HUVECs migration 11. Here, we report that mature VEGF‐D, both endogenously produced by HUVECs‐VEGF‐D^ΔNΔC^ and exogenously delivered by treatment with human recombinant VEGF‐D^ΔNΔC^, modulates the oxidant–antioxidant balance system in HUVECs. We have also examined the changes in the expression of proteins involved in the maintenance of redox homoeostasis, that is the effect of activation of the mammalian target rapamycin (mTOR) signalling pathway.

## Materials and methods

### Cell culture

Human vascular endothelial cells were cultured in M200 medium supplemented with Low Serum Growth Supplement (Life Technologies Corporation; Carlsbad, CA, USA). Before experiments, cells were starved in basal medium supplemented with 1% BSA or FBS.

### Adenovirus purification and cell transduction

For the proteomic 2D analysis, the HUVECs were transduced for 24 hrs with adenovirus vectors expressing the fully processed VEGF‐D (Ad‐VEGF‐D^ΔNΔC^) or green fluorescence protein (Ad‐GFP) as previously described 11.

### 2D gel electrophoresis and protein identification performed with LC‐MS/MS

The experimental procedure of 2D electrophoresis and data analysis were performed as described before with minor changes 13. Soluble proteins (75–200 μg) in the lysis buffer were mixed with a reswelling solution and loaded onto 24‐cm immobilized pH linear gradient (pH 4‐7) strip gels. The obtained MS/MS spectra were evaluated against the UniProt database with *Homo sapiens* taxonomy, and the status was reviewed (http://www.uniprot.org/) with the Protein Lynx Global Server Software (PLGS version 2.2.5, Waters Corporation, Milford, MA, USA).

### SDS‐PAGE and Western blotting analysis

The effect of VEGF‐D^ΔNΔC^ (R&D Systems Inc., Minneapolis, MN, USA) on proteins engaged in redox balance in HUVEC was evaluated using the antibodies to Prx2, Prx3, Prx6, CLIC1, CLIC4, SOD2, β‐actin and α‐tubulin (Abcam PLC, Cambridge, UK) and antibodies to mTOR and p‐mTOR (Ser2448) (Cell Signaling Technology, Danvers, MA, USA). All other antibodies, including the secondary antibodies conjugated with HRP, were from Santa Cruz Biotechnology, Inc. (Dallas, TX, USA). For mTOR inhibition experiments, an analogue of rapamycin‐temsirolimus (50 ng/ml, Cayman Chemical Company, Thallin, Estonia) was used 2 hrs before and during 24‐hrs stimulation of HUVECs by VEGF‐D.

The cells were solubilized in the lysis buffer (150 mM NaCl, 50 mM Tris‐HCl, pH 8.0) containing 5 mM EDTA and 1% NP‐40 for 30 min. on ice after scrapping and centrifuged (12,000×g, 4°C, 20 min.), and the total protein concentration was measured by BCA Protein Assay Reagent Kit (Thermo Scientific, Rockford, IL, USA). In phosphorylation studies, the bands intensities for the total and phosphorylated proteins were first normalized to the β‐actin and then compared to each other.

### Cell fractionation

Subconfluent cells were starved for 12 hrs in M200 supplemented with 1% FBS and treated with VEGF‐D (1 μg/ml) or VEGF‐A (25 ng/ml) for 6 hrs. Cytoplasmic and nuclear fractions were obtained according to manufacturer's protocol (NE‐PER Nuclear and Cytoplasmic Extraction Reagents, Thermo Scientific, Rockford).

### Reactive oxygen/nitrogen species production

Measurements of the intracellular reactive oxygen (ROS) and nitrogen species (RNS) production in the VEGF‐D‐treated HUVECs were performed by monitoring the oxidation of 2′,7′‐dichlorodihydrofluorescein diacetate (H2DCF DA) and 4‐amino‐5‐methylamino‐2′,7′‐difluorofluorescein diacetate (DAF‐FM DA), respectively (Thermo Scientific, Waltham, MA, USA). Assays were performed in the 96‐well microplates, in a HBSS solution containing 5.5 mM glucose, pH 7.4. After 12 hrs of HUVEC starvation, VEGF‐D was added for 4, 8, 12 and 24 hrs. The experimental medium was then replaced to HBSS with 5‐μM probe, and the fluorescence was monitored for 1 hr using Fluoroskan Ascent FL microplate reader.

### Measurement of the thiol group content in HUVECs

The total cellular thiol groups were assessed fluorometrically by conjugation with monobromobimane (mBrB) according to the manufacturer procedure for the ROS/RNS production assay (Thermo Scientific, Waltham). In the experiment, 5‐μM probe was used and the fluorescence of the bimane‐thiol conjugates was measured (Ex = 390 nm/Em = 460 nm) after 1 hr of incubation under cell culture conditions.

### Measurement of the total antioxidant capacity (TAC) of the VEGF‐D‐treated cells

Total antioxidant capacity was estimated by a modified ABTS*+ decolourization assay 14. After 12‐hrs starvation of HUVECs (M200 + 1% FBS), VEGF‐D was added for 12 or 24 hrs, and cell were lysed, by freeze–thaw cycles. Changes in the absorbance, at 414 nm, were registered after 10 sec. and 60 sec., which reflect to the reaction of ABTS*^+^ with (*i*) typical ‘fast‐reacting’ antioxidants, for example Vit C, GSH, uric acid and (*ii*) ‘slow‐reacting’ antioxidants, mainly protein tryptophan and tyrosine residues, respectively. All results are expressed in Trolox equivalents/mg of protein, measured after Lowry *et al*. 15.

### Cell viability assay

#### Cell treatment

The HUVECs were seeded at 20,000 cells/well in 96‐well plates. After 12 hrs of starvation of the HUVECs (M200+1% FBS), oxidative stress was induced *via* the addition of paraquat (N,N′‐dimethyl‐4,4′‐bipyridinium dichloride) or sodium hypochlorite (NaClO).

#### Resazurin reduction assay

After 24 hrs, the medium was removed and replaced by 0.0125 mg/ml of resazurin solution, for 2 hrs. Formed resorufin was quantified by measuring the relative fluorescence units, measured at Ex = 530 nm, Em = 590 nm.

#### Crystal violet assay

After the incubation, cells were fixed in 70% ethanol (30 min., RT). Than crystal violet staining solution (0.2% crystal violet in 20% methanol) was added for 10 min. After washing off the excess of dye with water, dissolving solution (0.1 M sodium acetate in 50% ethanol) was added and the absorbance was read at 570 nm.

### Statistical analysis

The data are expressed as means ± S.D. of at last three independent experiments. Statistical analysis was performed using two‐sided Student's *t*‐test; *P* < 0.001 (***),*P* < 0.01 (**) and *P* < 0.05 (*).

## Results

### Proteomic analysis of the HUVECs response to VEGF‐D^ΔNΔC^

**Figure 1:**
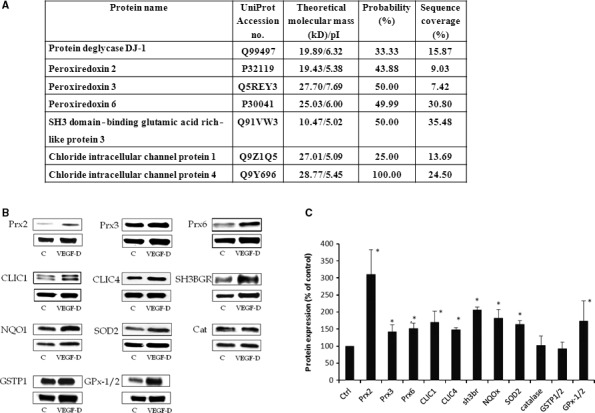
Effect of VEGF‐D on expression of proteins involved in control of redox balance in HUVECs. (**A**) Whole protein extracts were separated by 2D electrophoresis, and protein spots identified after silver staining to be changed by at least twofold in intensity were analysed by sequencing as described in Materials and methods. (**B**) HUVECs were incubated with rVEGF‐D^ΔNΔC^ (0.5 μg/ml), and proteins were extracted after 24 hrs with a lysis buffer and subjected to Western immunoblotting using antibodies to indicated proteins (representative images) (**C**) Semi‐quantification of blots. Data are presented as a mean ± S.D. of three independent experiments, and results were calculated as per cent of control. *P* < 0.05 (*) as compared to control (non‐stimulated cells).

Human vascular endothelial cells that were transduced with Ad‐VEGFD^ΔNΔC^ were compared with those transduced with Ad‐GFP (control vector). Changes in protein levels were determined with 2D electrophoresis. The expression of 32 proteins increased and expression of 14 proteins decreased by at least twofold in the cells that overexpressed VEGF‐D^ΔNΔC^ (Fig. S1). These proteins belong to the various cellular compartments: cytosolic, nuclear and mitochondrial (Table S1). Among the proteins significantly affected by VEGF‐D, we identified a group that is involved in oxidant–antioxidant homoeostasis signalling pathways (Fig. 1A).

The up‐regulation of proteins revealed by 2D electrophoresis (peroxiredoxins: Prx2, Prx3, Prx6, chloride intracellular channel proteins: CLIC1, CLIC4 and SH3 domain‐binding glutamic acid rich‐like protein 3) was also verified by Western blotting of cells incubated for 24 hrs with human recombinant VEGF‐D^ΔNΔC^ (rVEGF‐D^ΔNΔC^) at concentration of 0.5 μg/ml. The elevated levels of all proteins detected by the proteomic approach were positively confirmed (Fig. 1B and C). We have also analysed other proteins that are known to play a key roles in the cellular redox balance, such as superoxide dismutase 2 (SOD2), catalase (Cat), NAD(P)H dehydrogenase quinone 1 (NQO1), glutathione S‐transferase pi 1 (GSTP1) and glutathione peroxidase 1 (GPx‐1). As illustrated in Figure 1B and C, SOD2, NQO1 and GPx‐1 were significantly up‐regulated, whereas the levels of Cat and GSTP1 were not significantly altered upon treatment with rVEGF‐D^ΔNΔC^.

### Changes in the free radical production and total antioxidant capacity of HUVECs treated with VEGF‐D^ΔNΔC^

**Figure 2:**
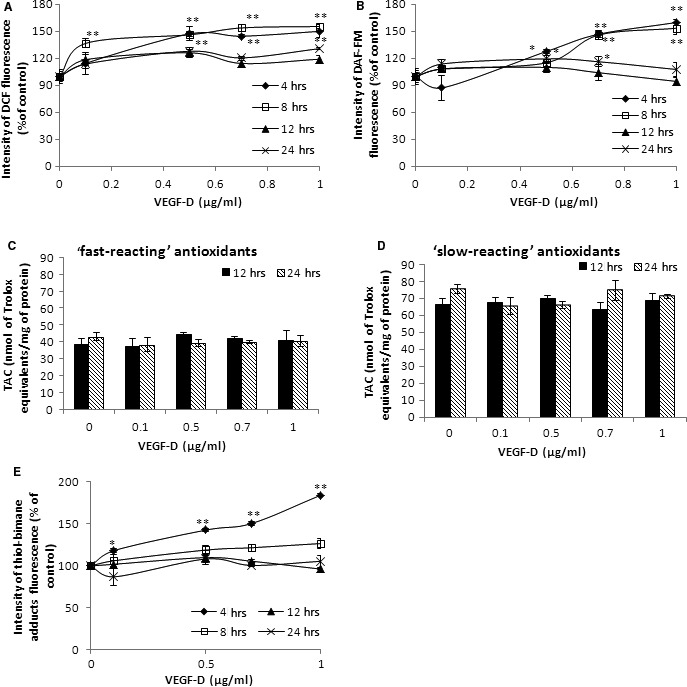
Changes in oxidant–antioxidant homoeostasis of HUVECs stimulated with VEGF‐D. HUVECs were incubated with rVEGF‐D^ΔNΔC^ (up to 1 μg/ml) for 4, 8, 12 and 24 hrs. (**A**) ROS and (**B**) RNS production was measured using oxidation of fluorescent probes H_2_DCF DA and DAF‐FM DA, respectively. (**C**,** D**) Total antioxidant capacity (TAC) of HUVECs upon VEGF‐D treatment was performed on freshly prepared cellular lysates and registered after 10 sec. (typical ‘fast‐reacting’ antioxidants) and 60 sec. (‘slow‐reacting’ antioxidants). (**E**) Changes in the thiol group content were detected by formation of the fluorescent bimane‐thiol adducts. Results are presented as (mean ± S.D.) of three independent experiments. *P* < 0.01 (**) and *P* < 0.05 (*).

Reactive oxygen/nitrogen species (ROS/RNS) production in HUVECs stimulated by rVEGF‐D^ΔNΔC^ was increased in time‐ and concentration‐dependent manner (Fig. 2A and B) to around 50% at the highest concentration of VEGF‐D (1 μg/ml). Increased ROS and RNS production was observed only after 4 and 8 hrs of incubation of the HUVECs with rVEGF‐D^ΔNΔC^. Prolonged incubation time (12 and 24 hrs), no longer significantly affected redox homoeostasis. Additionally, the total antioxidant capacity (TAC) estimated with the ABTS*+ decolourization assay was unchanged for both the fast‐ and slow‐reacting antioxidants (Fig. 2C and D). These data excluded the possibility of the induction of oxidative stress in the HUVECs by VEGF‐D and that conclusion was also confirmed by measurements of the thiol group content (Fig. 2E). Short‐duration treatment (4 hrs), of the HUVECs with increasing VEGF‐D concentration, resulted the increase in the SH content level, whereas the longer incubation did not show significant increase in the fluorescence intensity of the bimane‐thiol conjugates (Fig. 2E). These results indicate that a strongly reducing environment was correlated with the VEGF‐D concentration. Overall, the above data suggest that enhanced ROS/RNS production due to VEGF‐D treatment plays a messenger/regulatory role rather than a cytotoxic role.

### Cytoprotective effect of VEGF‐D against the oxidative stress conditions

**Figure 3:**
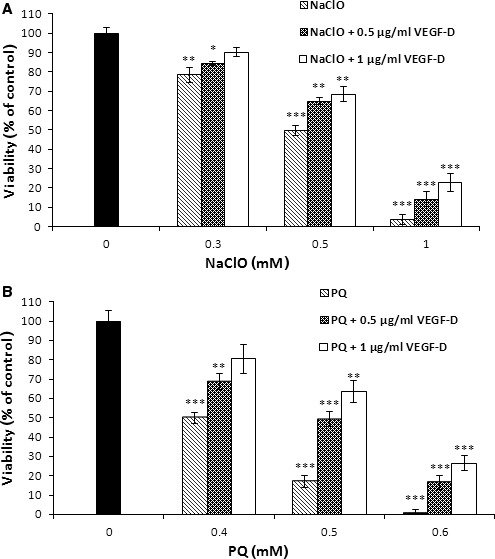
VEGF‐D enhances endothelial cells viability under oxidative stress conditions. HUVECs were cotreated with rVEGF‐D^ΔNΔC^ and oxidants: (**A**) hypochlorite or (**B**) paraquat for 24 hrs. After incubation, viability of ECs was evaluated using resazurin assay. Results are presented as (mean ± S.D.) of three independent and were calculated as per cent of control. *P* < 0.001 (***),*P* < 0.01 (**) and *P* < 0.05 (*) as compared to control (VEGF‐D and oxidants non‐treated HUVECs).

Treatment of HUVECs with increasing concentration of oxidants such as paraquat or sodium hypochlorite decreases their viability (Fig. 3, Fig. S2, light grey bars). The cotreatment with VEGF‐D had protective effects on cell viability and dependent on the concentrations of the growth factor (Fig. 3A and B, dark grey and white bars). The effect of VEGF‐D was observed for two different mechanisms of oxidative stress induction, that is the use of hypochlorite, which is an oxidant that is generated *in vivo* by the innate immune system and induces the misfolding of proteins, and paraquat, which is a pesticide that induces oxidative stress in mammals by participating in redox cycling with cellular enzymes to form the superoxide anion. These findings show the cytoprotective effect of VEGF‐D against oxidative stress conditions.

### VEGF‐D promotes the nuclear localization of mTOR

**Figure 4:**
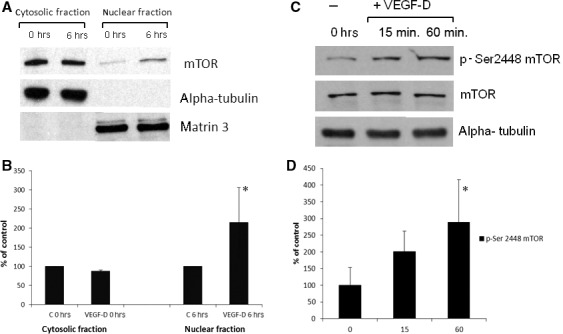
VEGF‐D induces cytoplasmic–nuclear shuttling of mTOR and affects its activity *via* stimulation of Ser2448 phosphorylation in HUVECs. (**A**) Cellular distribution of mTOR in cytoplasmic and nuclear fractions of HUVEC after 0 and 6 hrs of VEGF‐D treatment (1 μg/ml). (**B**) Semi‐quantification of blots. (**C**) HUVECs were incubated up to 60 min. with rVEGF‐D^ΔNΔC^. Whole cell extracts were probed with phospho‐Ser2448‐specific mTOR and with mTOR antibodies. (**D**) Semi‐quantification of blots. The bands intensities for the total and phosphorylated proteins were normalized to the β‐actin and then compared to each other. *P* < 0.05 (*).

Because VEGF‐D provides additional antioxidant support in HUVECs and given the role of mTOR pathway in the sensing and responses of cells to growth factors and stress conditions, we investigated the influence of rVEGF‐D^ΔNΔC^ on the mTOR kinase signalling pathway. First, we examined the expression of mTOR in whole‐cell lysates and in cytosolic and nuclear fractions of HUVECs cultured in full or experimental (1% FBS) media by Western immunoblotting. As expected, mTOR protein levels were significantly higher in the cytoplasm as compared to the nucleus. Because mTOR was detectable and unaffected by serum‐reduced conditions in both cytosolic and nuclear extracts (Fig. S3), we investigated the effects of VEGF‐D on both cytoplasmic and nuclear expression of mTOR. As shown in Figure 4A and B, VEGF‐D at the concentration of 1 μg/ml (the most effective concentration in oxidant–antioxidant haemostasis experiments, Fig. 2) promoted the nuclear localization of mTOR upon 6 hrs of stimulation. We have also compared the effects of VEGF‐D and VEGF‐A on the translocation of mTOR in the HUVECs to investigate the relative responses to VEGF‐D. The increase in nuclear and decrease in the cytoplasmic expression of mTOR after 6 hrs of treatment with VEGF‐D were lower than effect observed upon stimulation with VEGF‐A at the concentration of 25 ng/ml (Fig. S4). Off note, the purity of fractions was confirmed by analysing the cytosolic and nuclear markers, α‐tubulin and matrin 3, respectively.

Further, we have examined the expression of phosphorylated mTOR in the HUVECs after 15 and 60 min. of VEGF‐D stimulation (Fig. 4B). The significant increase in the phosphorylation of mTOR at Ser2448 was observed after treatment of the HUVECs with VEGF‐D for 60 min. (Fig. 4C and D). These results indicate that VEGF‐D regulates the mTOR pathway both by diminished the cytoplasmic mTOR fraction and by its phosphorylation at Ser2448.

### VEGF‐D‐dependent overexpression of antioxidant enzymes is rapamycin sensitive

**Figure 5:**
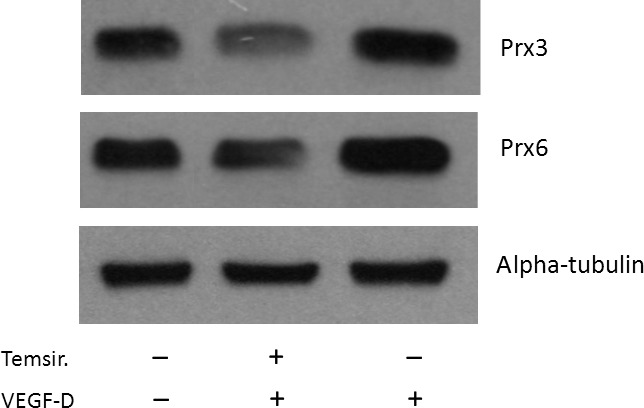
VEGF‐D up‐regulates expression of Prx3 and Prx6 in a rapamycin sensitive manner in HUVECs. HUVECs were incubated either with rVEGF^DΔNΔC^ (1 μg/ml) and temsirolimus (50 ng/ml, added 2 hrs before VEGF‐D stimulation)—lane 2 or with rVEGF^DΔNΔC^ only—lane 3. Proteins were extracted after 24 hrs with a lysis buffer and subjected to Western immunoblotting using antibodies to indicated proteins.

To further examine the biological outcome of mTOR nuclear translocation and phosphorylation, we have checked whether mTOR is required for the observed increase in the expression of proteins involved in the maintenance of redox homoeostasis. We show (Fig. 5) that 2 hrs pretreatment of HUVECs with temsirolimus, the selective inhibitor of mTOR (lane 2) decreased the expression of Prx3 and Prx6 as compared to HUVECs stimulated with VEGF‐D (lane 3).

## Discussion

Endothelial cells in tumour vessels display unusual characteristics in terms of survival and angiogenic properties due to the influence of diverse factors secreted by the tumour and other cells within the tumour microenvironment 16. A vast body of evidence indicates that inflammatory leucocytes contribute to cancer development directly *via* the release of growth and survival factors. Tumour‐associated macrophages, a major component of cancer‐related inflammation, were shown to produce pro‐angiogenic growth factors, such as VEGF‐D 17, 18. Tumour vessels express high levels of antioxidants to promote resistance to apoptosis and the immune response, mechanisms that are essential for tumour survival 19. The mechanisms by which VEGF‐D protects ECs under oxidative stress are currently unknown. We have suggested that the cytoprotective effects of VEGF‐D are due to its up‐regulation of endogenous cellular antioxidant systems that are capable of scavenging ROS. Based on our previous finding, which demonstrated the involvement of VEGF‐D in the transition of ECs from a quiescent to a proangiogenic/migratory phenotype 11, in present study, we show that VEGF‐D also up‐regulates a number of proteins engaged in the regulation of redox homoeostasis.

Proteins that belong to the antioxidant balance elements, that is peroxiredoxins: Prx2, Prx3, Prx6, chloride intracellular channel proteins: CLIC1, CLIC4 and SH3 domain‐binding glutamic acid rich‐like protein 3, were all affected by VEGF‐D. The increased expression of Prxs, CLIC1 and 4 and also protein deglycase DJ‐1, suggests that VEGF‐D has a protective effect against oxidative stress and may be antiapoptotic 20, 21, 22, 23.

Prxs are the family of proteins showing peroxidase activities that degrade H2O2 to H2O, thereby abolishing H2O2 toxicity. In addition to H2O2, peroxynitrite and lipid peroxides are also reduced by Prx enzymes 24. Interestingly, Prx2 has been found to be an essential antioxidant enzyme that prevents the oxidative inactivation of VEGF receptor‐2 (KDR) in the vascular tumour microenvironment 19. VEGF‐D up‐regulated three isoforms of Prxs that are localized mainly to cytosol (Prx2, 6) and mitochondria (Prx3). This observation suggests that VEGF‐D‐induced antioxidant barrier is not restricted to mitochondria.

Prxs are also the first line of defence removing H2O2 produced for cell signalling 24. They serve as a peroxide sensors to other protein and contribute to various redox signalling pathways that may modulate activity of phosphatases and tyrosine kinases or mitochondrial processes such as the TCA cycle, haem biosynthesis and OXPHOS in response to environmental changes 24, 25. Thioredoxin (Trx) was shown to be the biological donor of reducing equivalents for the catalytic function of Prx. VEGF‐D up‐regulated Prx6 that is the only mammalian 1‐Cys member of the Prx family and utilizes GSH instead of Trx as the physiological reductant to reduce H2O2 26. Moreover, apart from the cytoprotective roles, peroxiredoxins are frequently indicated as tumour markers 27, 28.

Atypical peroxiredoxin‐like peroxidase, protein DJ‐1, scavenges oxidative stress by oxidizing itself to a more acidic form and/or by increasing glutathione synthesis through an increase of glutamate‐cysteine ligase 29. It was observed that overexpression of DJ‐1 in animals or cultured cells prevents cell death, whereas knock‐down or knockout of DJ‐1 increases the susceptibility to oxidative stress 30.

VEGF‐D up‐regulates CLIC1 and CLIC4 that are reported to be strongly expressed in ECs 31. CLIC1 was also shown to be overexpressed in the tumour vasculature 32. The CLIC1 and CLIC4 knock‐downs in HUVECs resulted in reduced cell growth, modestly increased cell viability and inhibited network formation. The difference is that CLIC1 knock‐down only inhibits migration and CLIC4 knock‐down affects lumen formation. CLIC1 has been shown to induce invasion and proliferation of tumour as well as ECs and has a close relation with cytoskeletal proteins 33, 34, 35. CLIC proteins exhibit chloride ion channel activity and act like second messengers that can translocate to the cell membranes in response to modification of the basic cytoplasmic oxidative level. It has been shown that CLIC1 chloride current is necessary to support ROS production by NADPH oxidase (NOX) as its function is strictly dependent on the membrane potential 36.

Thus, our data demonstrate that VEGF‐D treatment may affect extracellular production of superoxide by NOX and may influence cell signalling through the regulated uptake of superoxide. These results are also in agreement with studies suggesting that VEGF may facilitate crosstalk between mitochondria and NOX during periods of increased ROS production 37.

Our observations raise the possibility that VEGF‐D is involved in endothelial protective actions during oxidative stress. These VEGF‐D effects were confirmed in our further analysis in the presence of the oxidative stress inducers NaClO and paraquat and are consistent with recent studies that found VEGF‐A to be instrumental for EC protection induced by ROS. Moreover VEGF‐D may up‐regulate expression of antioxidant system proteins not only for cell protection from oxidative stress but also to use them as secondary messengers 38, 39.

To further explore the effects of VEGF‐D on redox and antioxidant balance, we also studied the impact of the exogenous VEGF‐D on HUVECs as it is known that in solid tumours VEGF‐D is also secreted by both tumour cells and inflammatory cells present in tumour stroma 40. Therefore, the total level of VEGF‐D used should be higher than secreted by ECs alone. Concentration of VEGF‐D at range up to 1 μg/ml has been employed by other investigators, who demonstrated that at these concentrations of VEGF‐D activates intracellular signalling events and elicits endothelial cellular function 41, 42.

In the next sets of experiments, we analysed expression of other proteins that are known to play a key roles in the cellular redox balance, such as antioxidant enzymes: SOD, Cat, GPx‐1 and phase II detoxifing enzymes: NQO1 and GSTP1. As illustrated in Figure 1B and C, SOD2, NQO1 and GPx‐1 were significantly up‐regulated, whereas the levels of Cat and GSTP1 were not significantly altered upon treatment with VEGF‐D.

Up‐regulated SOD2 protein expression suggests increased removal of superoxide and H2O2 in VEGF‐D‐treated HUVEC. In particular, because of its subcellular localization, mitochondrial SOD2 serves as a critical antioxidant defence against superoxide produced by respiration and prevents its reaction with nitric oxide that leads to the formation of highly deleterious peroxynitrite species [43]. It was demonstrated that SOD2 overexpression protects mitochondrial respiratory function and blocks apoptosis induction during heart ischaemia reperfusion injury and attenuates mitochondrial ROS generation, intracellular lipid peroxidation and cell death [44, 45]. Thus, the up‐regulation of mitochondrial SOD2 by VEGF‐D represents an important mechanism for its protection of ECs against oxidative cytotoxicity resulting from mitochondrial oxidative stress. It should be underlined, however, that ROS produced by mitochondria in ECs serves as an intracellular signalling factors and VEGF‐D may also be involved in regulation of their function [46].

Glutathione peroxidase 1 is a crucial antioxidant enzyme that like Prxs is involved in preventing of the harmful accumulation of intracellular H2O2. It is present in all components of cells; found in cytosolic and mitochondrial compartments [25, 47]. Similar to Prxs, the capacity of GPxs to transfer their oxidation state to other proteins, in this case through the S‐glutathionylation of cysteine residues on the recipient proteins, is thought to be part of signalling systems [48].

However, Prxs and GPxs play different roles in detoxifying ROS. The high activity and abundance of Prx is important for converting low levels of ROS, and as such are more likely to act as the final acceptor of low levels of ROS associated with signalling [49]. Thus, Prxs would be most beneficial under physiological conditions where low levels of ROS must be accurately coordinated; GPxs can provide the added support required for detoxifying ROS at higher concentrations [48].

Along with simultaneous up‐regulation of SOD2 and peroxide clearance enzymes, we observed no increase in Cat level during VEGF‐D treatment. Its up‐regulation by H2O2 would be expected in our experiments. However, Cat is peroxisomal enzyme and innumerable studies have documented its activation by peroxisomal proliferators, such as clofibrate and other fibrates or by oxidized fatty acids [50, 51]. It was shown that overexpression of mitochondrial Cat also inhibited VEGF‐induced cell migration [52].

Glutathione peroxidase 1 has been found to be more effective than Cat at removing intracellular peroxides under many physiological conditions, [53] and thus, VEGF‐D‐induced up‐regulation of GPx‐1 in HUVECs may serve as alternative to Cat.

Similarly, VEGF‐D‐induced NQO1 expression that may compensates for SOD1 activity. Predominantly located in the cytoplasm, NQO1 has been reported, in addition to its catalytic role in reduction of quinones, to scavenge superoxide directly, albeit less efficiently than SOD. This activity especially is important in tissues with low SOD. In cardiovascular cells, where expression of NQO1 is high and that of SOD is relatively low, induction of NQO1 was found to correlate with increased superoxide scavenging, whereas its inhibition led to a decrease in superoxide scavenging [54, 55].

VEGF is an important mediator of EC migration and angiogenesis. One important way in which VEGF stimulates EC migration is through production of ROS [52].

The ROS‐mediated migration may be a consequence of oxidative molecules that modify activity in several protein kinase and phosphatases [56]. Other studies demonstrated the requirement of low levels of ROS, including H_2_O_2_ and superoxide for ECs migration, but the specific proteins that are targeted and the signalling that is affected are not well defined [52]. As our previous finding demonstrated the involvement of VEGF‐D in the transition of ECs from a quiescent to proangiogenic/migratory phenotype 11 in current studies, we hypothesized that VEGF‐D generates ROS and uses them as a downstream signal mediator in HUVECs.

Our data show that ROS and RNS production following VEGF‐D treatment was increased in time‐ and concentration‐dependent manner. Moreover, the increased levels of ROS and RNS were transient and exhibited signs of a regulatory role; no change in total antioxidant capacity was found. Interestingly, the increase in ROS production did not lead to a decrease in the level of total thiols in the cells, which demonstrated the limited extent of VEGF‐D‐induced oxidative changes. At the same time, there was an efficient antioxidant defence that enabled the maintenance of the stress within the scope of signalling and not bulk damage. These results demonstrated that VEGF‐D‐induced ROS act as physiological signal transduction messengers and may contribute to the function of VEGF‐D in ECs. Furthermore, the ROS production due to HUVECs stimulation with VEGF‐D corresponds with VEGF‐A‐dependent ROS production and thus illustrates an alternative function of VEGF‐D in angiogenesis [57, 58].

To gain further insight into regulatory role of VEGF‐D, we analysed its effects on the mTOR signalling pathway as mTOR is known to act as a sensor that integrates extracellular and intracellular events and coordinates growth and proliferation. Mammalian target rapamycin is a multifaceted kinase that employs a number of effectors to exert its biological functions.[59]. Subcellular localization may be a general principle used by mTOR to govern cellular sensitivity to signal events and enact precise spatial and temporal control of cell growth [60]. We detected by Western blotting that VEGF‐D promoted the nuclear localization of mTOR; however, the observed effect of the mTOR nuclear shuttling was slightly lower in comparison to the effect of VEGF‐A. This confirms that efficacy, kinetics and range of response in ECs to VEGF‐D differ markedly from that induced by VEGF‐A 41. It has been found that the nuclear shuttling of mTOR is critical for the signalling to its cytoplasmic targets p70S6K1 and 4E‐BP1 and regulates cap‐dependent initiation of translation [61]. Mammalian target of rapamycin becomes nuclear in HEK293 cells treated with leptomycin B and inhibition of mTOR nuclear export coincides with diminished S6K activation and 4E‐BP1 phosphorylation [62]. The nuclear localization of mTOR has been also observed in rhabdomyosarcomas (Rh30 and Rh41), human fibroblasts (IMR90), multiple myeloma cells and colon carcinoma cells (HCT8) and was frequently implicated in resistance to anticancer therapies, including tyrosine kinase inhibitors, radiation and cytotoxic drugs. [63, 64].

In addition to modulation of mTOR nuclear shuttling in ECs, VEGF‐D appears to promote mTOR‐Ser2448 phosphorylation. We observed the significant increase in the phosphorylation of mTOR at Ser2448 after treatment of the HUVECs with VEGF‐D. As indicated in the literature, Akt as well as p70S6 kinase and mTOR itself are engaged in this process [65–67].

The above results suggest that the VEGF‐D activation of mTOR signalling can positively regulate protein synthesis and modify the expression of elements comprising the oxidant–antioxidant balance system. We confirmed this hypothesis providing evidence that temsirolimus, the well‐established inhibitor of mTOR, decreased the expression level of key antioxidants induced by VEGF‐D, that is cytosolic Prx6 and mitochondrial Prx3.

However, it is possible that mTOR's role in translation initiation involves more than p70S6K1 activation and 4EBP1 phosphorylation. Besides regulation of translation, the mTOR pathway has also been implicated in the control of many other cellular processes, such as ribosome biogenesis or transcription [68]. Thus, the optimal VEGF‐D‐dependent biological outcome would be the result of balance among several mTOR functions and further investigation should be undertaken to determine it.

In summary, our present study indicates that VEGF‐D functions not only as a stimulator of the proangiogenic phenotype of ECs but also as a modulator of the antioxidant potential of cells that ensures their survival in the tumour microenvironment. Although VEGF‐A was shown to be the most important proangiogenic factor in almost all solid tumours, its down‐regulation does not result in the complete inhibition of tumour angiogenesis. The development of blood vessels may occur as a result of VEGF‐D up‐regulation. Tumour cells show ‘adaptive ability’ to antiangiogenic therapy; that is, not only do tumours have multiple pathways for angiogenic signalling, but they also have the ability to call on the secondary pathways when the primary pathway is inhibited 9. Our results are in agreement with this finding and indicate that VEGF‐D is a plausible alternative mediator of angiogenesis and supports the activity of VEGF‐A.

## Specific contributions of the authors

IP‐P, AB: designed and performed the research study and wrote the paper. ES‐M and WK: performed the experiments. JB analysed the data and participated in the writing of the final manuscript.

## Conflict of interest statement

The authors have no conflict of interests to declare.

## Supporting information


**Figure S1** 2D gel electrophoresis of endothelial cells transduced with Ad‐VEGF‐DΔNΔC.
**Table S1** Changes of protein expression in HUVECs transduced with Ad‐VEGF‐DΔNΔC.
**Figure S2** VEGF‐D enhances endothelial cells viability under oxidative stress conditions.
**Figure S3** Western blot gel documents data showing the cytoplasmic and nuclear expression of mTOR and its’ resistance to serum starvation in both cytosolic and nuclear extracts.
**Figure S4** Cytoplasmic‐nuclear shuttling of mTOR in HUVECs after VEGF‐D or VEGF‐A treatment.
**Figure S5** Model of ROS control by VEGF‐D.
