# Supplementary material for: Vascular endothelial growth factor‐D modulates oxidant–antioxidant balance of human vascular endothelial cells
Source: J Cell Mol Med. 2016 Dec 13;21(6):1139–49. doi: 10.1111/jcmm.13045 (PMC5431135; doi:10.1111/jcmm.13045)
Supplement: Supplementary file 1 — Figure S1 2D gel electrophoresis of endothelial cells transduced with Ad‐VEGF‐DΔNΔC. Table S1 Changes of protein expression in HUVECs transduced with Ad‐VEGF‐DΔNΔC. Figure S2 VEGF‐D enhances endothelial cells viability under oxidative stress conditions. Figure S3 Western blot gel documents data showing the cytoplasmic and nuclear expression of mTOR and its’ resistance to serum starvation in both cytosolic and nuclear extracts. Figure S4 Cytoplasmic‐nuclear shuttling of mTOR in HUVECs after VEGF‐D or VEGF‐A treatment. Figure S5 Model of ROS control by VEGF‐D. [file JCMM-21-1139-s001.doc]

**SUPPORTING INFORMATION**

**Vascular endothelial growth factor-D modulates the oxidant-antioxidant balance of human vascular endothelial cells and induces mTOR shuttling**

Izabela Papiewska-Pajak^1^, Aneta Balcerczyk^2^, Emilia Stec-Martyna^3^, Wiktor Koziołkiewicz^4^, Joanna Boncela^1^*

^1^ Institute of Medical Biology, Polish Academy of Science, Lodz, Poland ^2^ Department of Molecular Biophysics, University of Lodz, Poland ^3^ Central Scientific Laboratory Medical University ^4^ Department of Molecular and Medical Biophysics, Medical University of Lodz, Poland

*corresponding author

Institute of Medical Biology, Polish Academy of Science, 106 Lodowa Street, 93-232 Lodz, Poland

E-mail:

Tel. (+48) 422723633

Fax (+48) 422723630

**
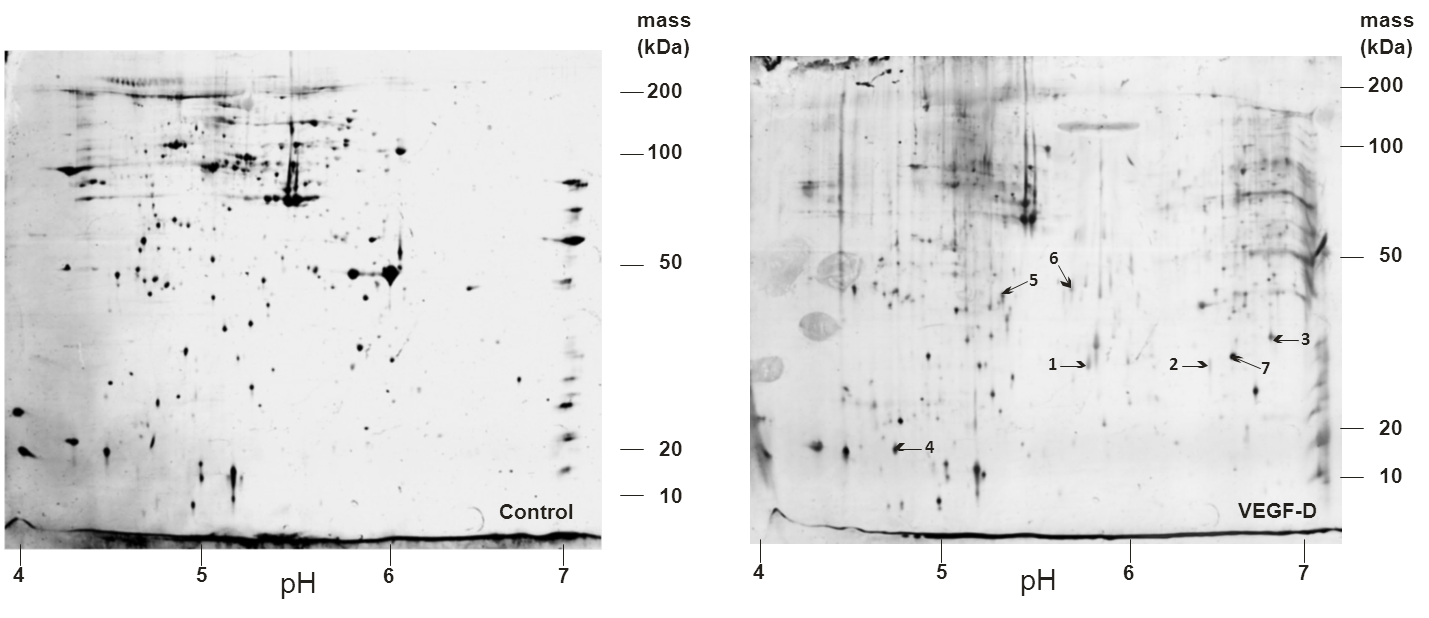
**

**Figure S1.** **2D gel electrophoresis of endothelial cells transduced with Ad-VEGF-DΔNΔC**

Representative 2D gel electrophoresis of endothelial cell lysates from cells transduced with Ad-GFP or Ad-VEGF-DΔNΔC. The lysates were separated using pH gradient ranged from 4.0 to 7.0 in the first direction and 12.5% gels in the second direction. The proteins were detected by silver staining. Arrows indicate the proteins involved in maintaining oxidant-antioxidant homeostasis, which content increased at least two-fold when both type of cells were compared: 1- Prx2, 2 - Prx3, 3 – Prx6, 4 - SH3 domain binding glutamic acid rich like protein 3, 5 - CLIC1, 6 - CLIC4, 7 – DJ-1.

**Table S1. Changes of protein expression in HUVECs transduced with Ad-VEGF-DΔNΔC**

Whole protein extracts were separated by 2D electrophoresis and protein spots identified after silver staining to be changed by at least 2-fold in intensity were analyzed by sequencing as described in Material and Methods. Sequence coverage - The percentage of the protein sequence covered by identified peptides.

| **Proteins UPREGULATED in HUVECs overexpressed VEGF-D**ΔNΔC | | | | |
| --- | --- | --- | --- | --- |
| **Protein name** | **UniProt Accession no.** | **Theoretical molecular mass (kDa)/pI** | **Probability**  **(%)** | **Sequence coverage (%)** |
| Vimentin | P48670 | 51.85/4.93 | 18.54 | 9.30 |
| Myosin light polypeptide 3 | P16409 | 22.16/5.03 | 99.99 | 10.00 |
| Myosin light polypeptide 6 | P60662 | 16.93/4.56 | 16.67 | 31.78 |
| Myosin regulatory light chain 2 | Q5RC34 | 19.79/4.65 | 14.89 | 29.82 |
| Cofilin 1 | P23528 | 18.52/8.16 | 14.28 | 18.10 |
| LIM and SH3 domain protein 1 | Q3B7M5 | 29.67/6.61 | 17.14 | 9.56 |
| Stathmin | P16949 | 17.30/5.75 | 24.95 | 23.48 |
| Protein deglycase DJ-1 | Q99497 | 19.89/6.32 | 33.33 | 15.87 |
| Peroxiredoxin 2 | P32119 | 19.43/5.38 | 43.88 | 9.03 |
| Peroxiredoxin 3 | Q5REY3 | 27.70/7.69 | 50.00 | 7.42 |
| Peroxiredoxin 6 | P30041 | 25.03/6.00 | 49.99 | 30.80 |
| SH3 domain binding glutamic acid rich like protein 3 | Q91VW3 | 10.47/5.02 | 50.00 | 35.48 |
| Calpain small subunit 1 | P06813 | 28.24/5.16 | 99.99 | 14.28 |
| Chloride intracellular channel protein 1 | Q9Z1Q5 | 27.01/5.09 | 25.00 | 13.69 |
| Chloride intracellular channel protein 4 | Q9Y696 | 28.77/5.45 | 100.00 | 24.50 |
| Annexin A1 | P04083 | 38.71/6.57 | 50.00 | 17.34 |
| Annexin A5 | Q5R1W0 | 35.94/4.93 | 33.33 | 12.80 |
| Tumor protein D54 | O43399 | 22.24/5.26 | 100.00 | 16.50 |
| Endoplasmic reticulum resident protein 29 | P30040 | 28.99/6.77 | 100.00 | 31.41 |
| Ubiquitin conjugating enzyme E2 N | P61088 | 17.14/6.13 | 16.74 | 13.81 |
| Galectin 1 | P09382 | 14.71/5.30 | 100.00 | 22.22 |
| Triosephosphate isomerase | P00939 | 26.62/7.09 | 25.91 | 30.64 |
| Nucleoside diphosphate kinase A | P15531 | 17.15/5.81 | 49.99 | 23.68 |
| ATP synthase subunit d, mitochondrial | O75947 | 18.49/5.21 | 100.00 | 21.73 |
| 39S ribosomal protein L12, mitochondrial | P52815 | 21.35/9.05 | 99.99 | 16.16 |
| Biliverdin reductase A | P53004 | 33.43/6.06 | 100.00 | 9.45 |
| Sorcin | Q5R4U9 | 21.68/5.32 | 50.00 | 11.11 |
| Alpha synuclein | P61143 | 14.47/4.72 | 12.50 | 22.14 |
| Transaldolase | P37837 | 37.54/6.36 | 100.00 | 13.94 |
| Translation initiation factor 3 subunit 1 | Q0VCU8 | 28.95/4.72 | 26.17 | 8.90 |
| Hepatoma derived growth factor | Q8VHK7 | 26.49/4.78 | 25.52 | 8.20 |
| Eukaryotic translation initiation factor 5A | Q26571 | 54.95/10.03 | 83.63 | 15.38 |
| Proteins DOWNREGULATED in HUVECs overexpressed VEGF-DΔNΔC | | | | |
| Protein name | UniProt Accession no. | Theoretical molecular mass (kDa)/pI | Probability  (%) | Sequence coverage (%) |
| T complex protein 1 subunit theta | P50990 | 59.62/5.41 | 100.0 | 3.75 |
| T complex protein 1 subunit epsilon | P48643 | 59.67/5.44 | 100.0 | 17.00 |
| CCT8 protein | Q7Z759 | 54.11/5.17 | 72.5 | 37.22 |
| Calreticulin | P27797 | 48.14/4.29 | 100.0 | 21.34 |
| Proteasome subunit beta type 4 | P28070 | 29.20/5.70 | 100.0 | 5.30 |
| Proteasome activator complex subunit 1 | Q06323 | 28.72/5.78 | 100.0 | 24.89 |
| Proteasome activator complex subunit 3 | P61289 | 29.50/5.69 | 87.0 | 15.35 |
| Proliferating cell nuclear antigen | P17918 | 28.78/4.66 | 33.3 | 9.19 |
| Annexin A4 | P09525 | 35.89/5.83 | 100.0 | 33.85 |
| Annexin A6 | P08133 | 75.87/5.41 | 100.0 | 18.42 |
| L-Lactate dehydrogenase | Q5U077 | 36.64/5.71 | 100.0 | 31.43 |
| Histone H2A | Q8SSG3 | 14.01/9.67 | 100.0 | 14.06 |
| Protein disulfide isomerase A3 | P30101 | 56.78/5.98 | 100.0 | 24.95 |
| Prohibitin | P35232 | 29.80/5.57 | 99.9 | 22.42 |


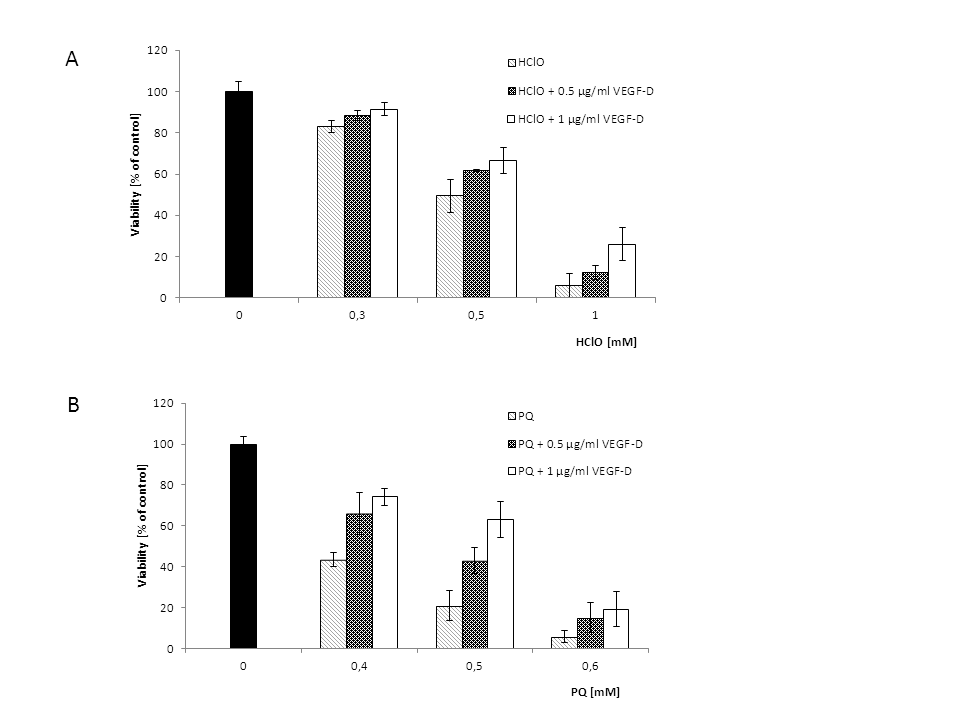


**Figure S2. VEGF-D enhances endothelial cells viability under oxidative stress conditions**

HUVECs were co-treated with rVEGF-D^ΔNΔC^ and oxidants: (A) hypochlorite or (B) paraquat for 24 hrs. After incubation, viability of ECs was evaluated using Crystal violet assay.

**
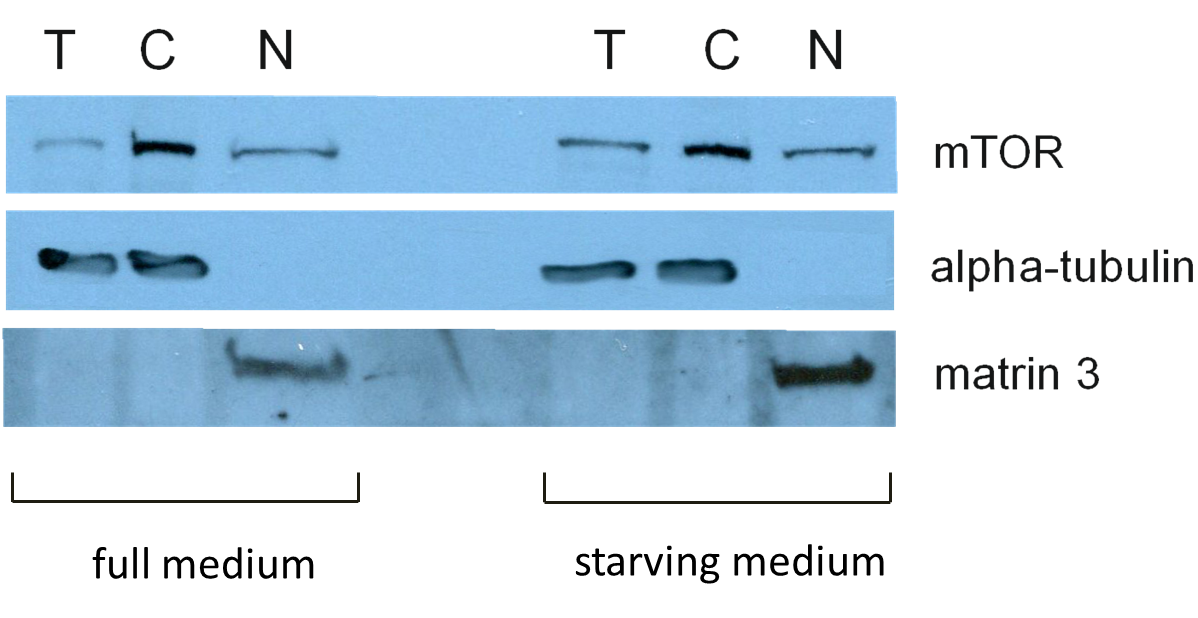
**

**Fig. S3.** Western blot gel documents data showing the cytoplasmic and nuclear expression of mTOR and its’ resistance to serum starvation in both cytosolic and nuclear extracts.

**
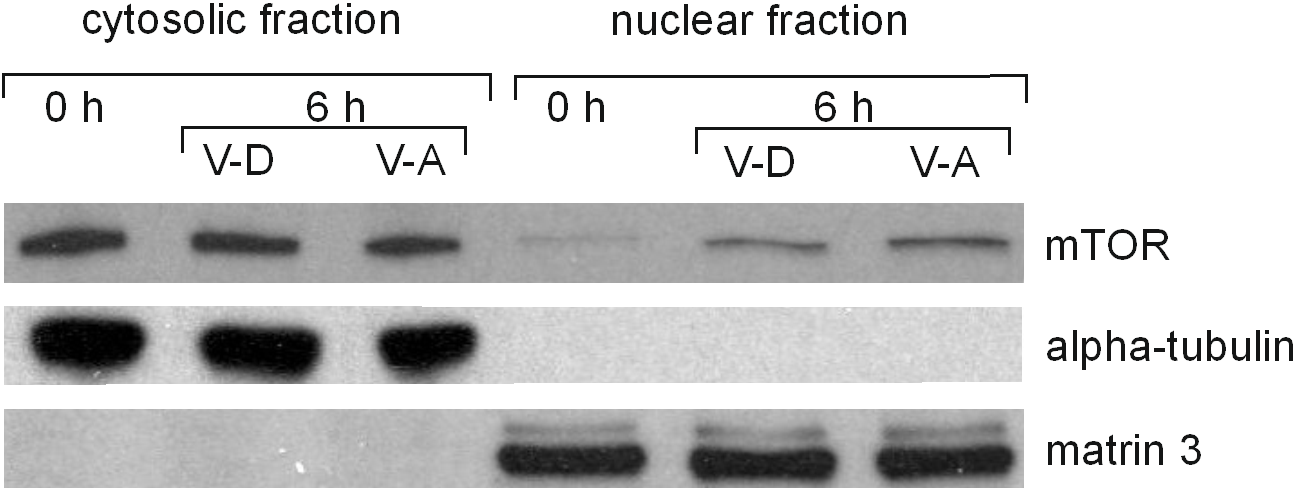
**

**Figure S4. Cytoplasmic-nuclear shuttling of mTOR in HUVECs after VEGF-D or VEGF-A treatment**

Comparison of mTOR levels in cytosolic and nuclear fractions of HUVEC after 6 hrs of treatment with VEGF-D and VEGF-A by western blot analysis.


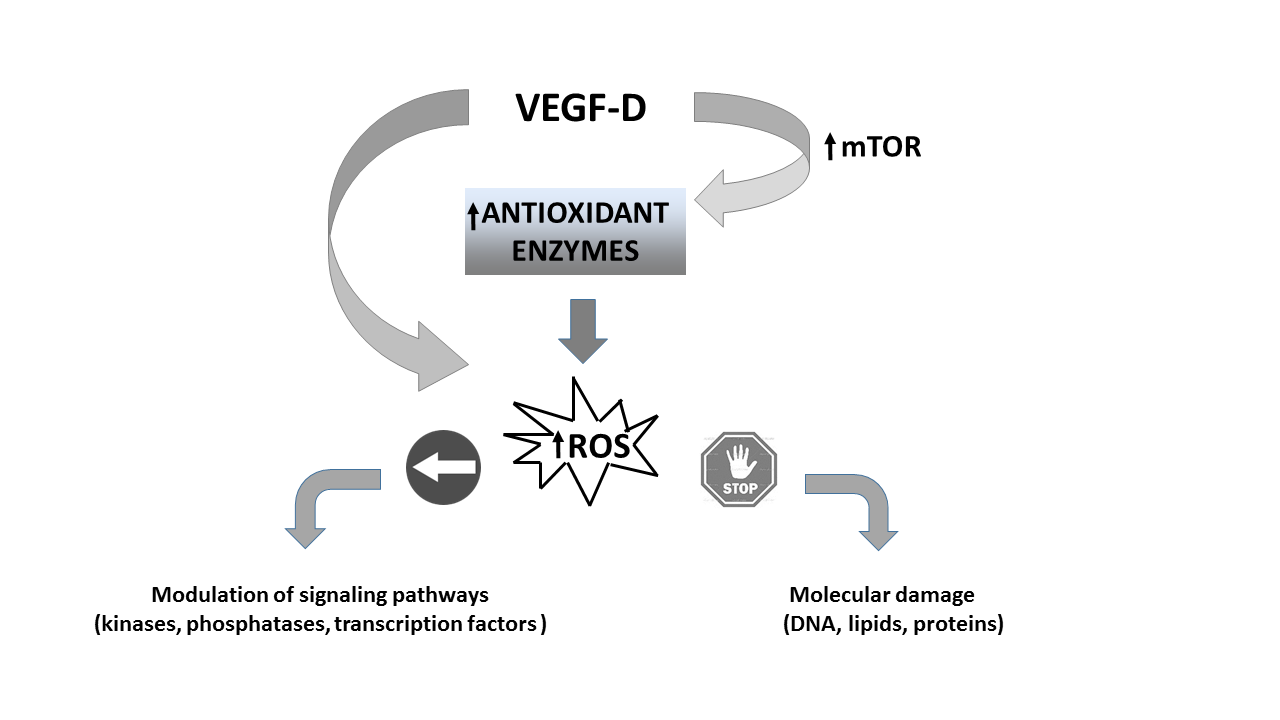


**Figure S5: Model of ROS control by VEGF-D.**

VEGF-D upregulates expression of the antioxidants enzymes. This effect is rapamycin sensitive. The VEGF-D activation of mTOR signalling positively regulate protein synthesis of elements comprising the oxidant-antioxidant balance system. The increase in expression of the antioxidant enzymes leads to efficient antioxidant defence. Thus, VEGF-D keeps ROS within the scope of signalling and prevents from bulk damage.
